# Psychological distress and health-related quality of life among women with breast cancer: a descriptive cross-sectional study

**DOI:** 10.1007/s00520-021-06763-z

**Published:** 2021-12-23

**Authors:** Nitikorn Phoosuwan, Pranee C. Lundberg

**Affiliations:** 1 Department of Public Health and Caring Sciences, Faculty of Medicine, Uppsala University, BMC, Husargatan 3, Box 564, 751 22 Uppsala, Sweden; 2 Department of Community Health, Faculty of Public Health, Kasetsart University Chalermphrakiat Sakonnakhon Province Campus, Sakon Nakhon, Thailand

**Keywords:** Anxiety, Depressive symptoms, Health-related quality of life, Support, Women with breast cancer

## Abstract

**Purpose:**

Breast cancer is the most common type of cancer found in women in Sweden and worldwide. Treatment leads to increased survival of patients, but they are at risk to experience psychological distress, including anxiety and depressive symptoms, and decreased health-related quality of life (HRQoL). This study investigated the relationship between psychological distress and HRQoL and related factors among women with breast cancer in Sweden.

**Methods:**

This descriptive cross-sectional study was conducted in Sweden. A total of 481 women with breast cancer answered voluntarily a questionnaire about sociodemographic and support factors, psychological distress, and HRQoL. Data were subjected to Pearson’s correlation and linear regression analyses.

**Results:**

Psychological distress was related to HRQoL in terms of body image, future perspective, side effects of systemic therapy, breast symptoms, arm symptoms, and hair loss. Women with lower age were associated with increased symptoms of anxiety, while those having undergone breast reconstruction were associated with increased symptoms of depression. Breast reconstruction and chemotherapy worsened body image, low support from partner decreased sexual functioning and enjoyment, and low support from physicians and nurses worsened future perspective, side effects of systemic therapy, breast symptoms, and indignation about hair loss.

**Conclusions:**

Psychological distress was correlated with the HRQoL. Increased support from physicians, nurses, and husband/partner may increase the HRQoL among women with breast cancer. Breast cancer treatments such as breast reconstruction and chemotherapy were factors that decreased the psychological distress and increased the HRQoL.

## Background

Worldwide, cancer is a major cause of morbidity and mortality; about 15 million new cases were found in 2012, and eight million people died from diseases related to cancer [1]. Breast cancer is the most common type of cancer among women, and more than 12 percent of women are diagnosed with breast cancer [2]. In 2018, Sweden had an age-adjusted rate of breast cancer of 89.8 per 100,000 [3, 4]. Although improved diagnostics and treatments lead to an increased survival rate among patients with breast cancer [5], 1,400 Swedish women died due to breast cancer in 2006 [4]. After diagnosis, women with breast cancer are at high risk to experience psychological distress [6, 7] and may have decreased health-related quality of life (HRQoL) [8].

Psychological distress is a state of emotional suffering commonly characterized by symptoms of depression and anxiety [9]. More than 25% of women with breast cancer suffer from such symptoms [7, 10]. They are more likely to have suicidal thoughts than the general population [11]. Some factors are related to the psychological distress among them. Some factors are related to the psychological distress among them. Women with breast cancer living in a rural area, being Christian, or having traits of anxiety at the time of diagnosis are associated with psychological distress [7, 12]. Psychological distress may be a forerunner to mental, physical, and emotional exhaustion in a country with high a incidence rate of breast cancer like Sweden [9, 13]. There is a need of an investigation of factors that help to avoid mental, physical, and emotional chaos in patients with breast cancer [9].

HRQoL refers to an individual’s perception of his or her position in life, covering independence; physical, psychological, and social relations; and environmental and spiritual dimensions [14]. HRQoL has been acknowledged as an important outcome for patients with cancer [15]. HRQoL among women with breast cancer is often poorer in comparison with women in the general population regarding social and emotional functioning [16, 17]. Sociodemographic characteristics, including low age, low education, financial problems, and occupation, could be factors associated with low HRQoL [10, 13, 18, 19]. Chemotherapy treatment, time since diagnosis, and lack of support from family and friends are also associated with lower HRQoL among women with breast cancer [8, 10, 13, 18]. Lack of emotional support from professional counselors in hospitals leads to psychological distress among cancer patients, who need support also from family and friends [20].

In addition, psychological distress and stress are correlated with lower HRQoL [8, 19, 21]. Although important, these factors are often neglected or under-recognized [22]. Therefore, psychological distress and the HRQoL among women with breast cancer need to be investigated [22]. The aim of this study is to investigate the relationship between psychological distress and the HRQoL among women with breast cancer in Sweden. It is also to investigate different factors that affect psychological distress and the HRQoL such as sociodemography, treatments, and support (e.g., from healthcare personnel in hospitals, husband/partner, family, and friends).

## Materials and methods

### Study setting and design

A descriptive cross-sectional study was carried out in three cities in Sweden: Uppsala, Gävle, and Falun.

### Participants

Based on the Regional Cancer Centre (RCC) in Uppsala and Örebro, registered women were invited to participate in the study. The inclusion criteria were: Women who (1) had been diagnosed with breast cancer at least 1 year before data collection to ensure unchanged diagnosis; (2) were at least 18 years old; (3) lived in Uppsala, Gävle, or Falun; and (4) were willing to participate. Women who reported a history of mental disorder or dementia were excluded. In total, 481 out of 975 eligible women with breast cancer agreed to participate in the study.

### Instruments

The use was made of a questionnaire containing four parts: (1) sociodemographic characteristics, (2) support, (3) psychological distress, and (4) HRQoL. Sociodemographic characteristics concerned age, marital status, education, religion, belonging to a cultural/ethnic minority, having an underlying disease, duration of diagnosed breast cancer, methods of treatment (i.e., chemotherapy, radiotherapy, Herceptin, and hormone therapy).

The part support concerned six sources of support, viz., physicians, nurses, the Internet, husband/partner, family members and friends, and the patient’s institution. It was created by PCL. Each source comprised nine questions, each of which gave a score of zero if the answer was “no” and one if the answer was “yes.” Therefore, each source could give a total score ranging from zero to nine, and a higher total score indicated more support. This part had a Cronbach’s alpha coefficient of 0.89.

The part psychological distress comprised of anxiety and depressive symptoms. Anxiety and depressive symptoms were measured by use of the Hospital Anxiety and Depression Scale [23]. The scale had 14 items divided into 2 subscales: one measured anxiety (HADS-A) and the other measured depressive symptoms (HADS-D). Each subscale had seven items with a four-Likert scale. The total possible score for each subscale ranged from zero to 21, and a higher score indicated more symptoms. HADS-A and HADS-D had Cronbach’s alpha coefficients of 0.89 and 0.84, respectively, for Swedish women with breast cancer [13].

HRQoL was measured using the European Organization for Research and Treatment of Cancer Breast Cancer-Specific Quality of Life Questionnaire (QLQ-BR23) [24]. It is a disease-specific questionnaire with 23 questions, each of which had four options assigned by a number (not at all = 1, a little = 2, quite a bit = 3, and very much = 4). It assessed eight dimensions: body image (BRBI), sexual functioning (BRSEF), sexual enjoyment (BRSEE), future perspective (BRFU), side effects of systemic therapy (BRST), breast symptoms (BRBS), arm symptoms (BRAS), and indignation by hair loss (BRHL) [25]. All dimensions were transformed to 100-percent scores, and higher scores indicated the lower quality of life. This questionnaire was translated to Swedish and tested before data collection among other breast cancer patients with an acceptable Cronbach’s alpha score in each sub-scale [13].

### Procedure

The heads and nurses of clinics of surgery/oncology and plastic surgery in Uppsala, Gävle, and Falun were informed about the study by one of the researchers (PCL). They asked questions until they found that everything about the study was clear. In this way, the nurses became able to answer questions if the participants would ask. Finally, the heads of the clinics gave permission to conduct the study. Written information about the study and its purpose was sent by ordinary mail together with a consent letter and a questionnaire to the eligible women. They were assured of their anonymity and of confidentiality, and they were told that they could drop out at any time. The Declaration of Helsinki for medical research was fulfilled. The women who agreed to participate in the study signed a consent letter, responded to the questionnaire, and returned these documents in a stamped envelope. Women who did not wish to participate in the study returned the documents without filling in any information. A reminder was sent twice by post (after two weeks and one month) to women who had not returned the envelope in due time.

**Fig. 1:**
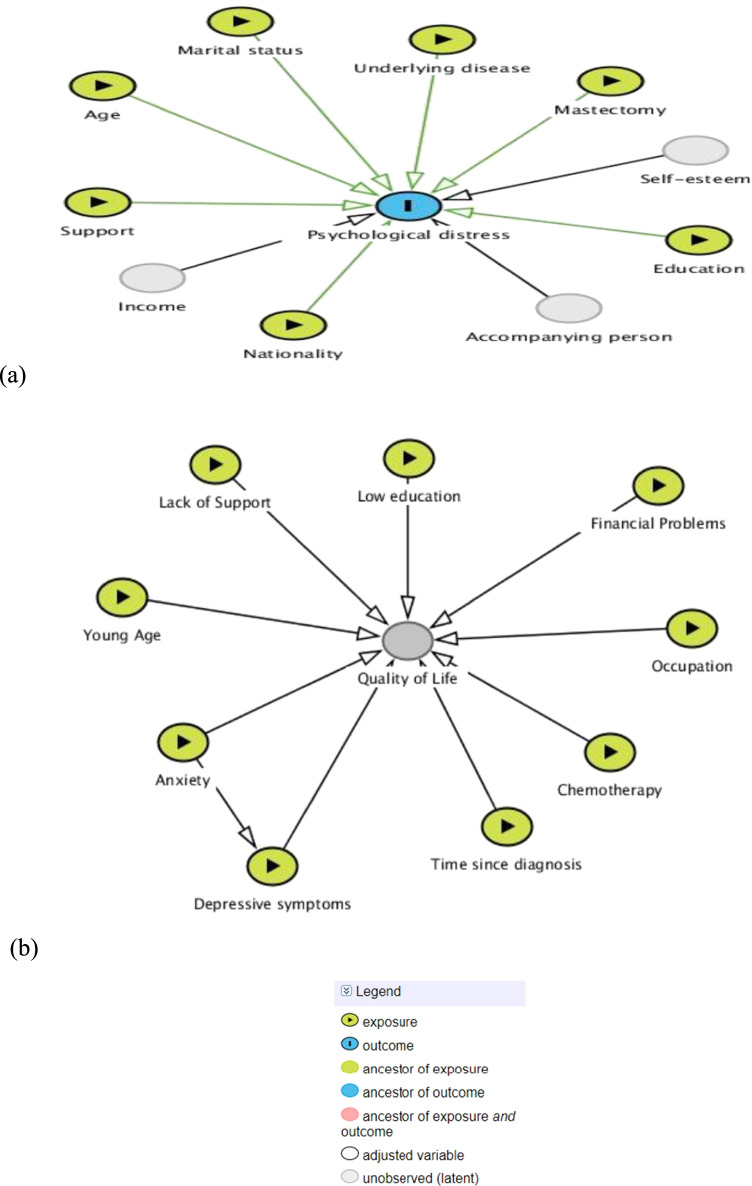
Directed acyclic graphs for possible risk factors associated with **a** psychological distress and **b** quality of life among cancer patients

Directed acyclic graphs (DAGs) [26] were constructed based on previous studies in order to demonstrate what factors were associated with psychological distress [27–29] and with HRQoL [13]. See Fig. 1a and b.

### Analyses

We analyzed data using descriptive and inferential statistics. Descriptive statistics summarized sociodemographic characteristics, social support, HADS-A scores, HADS-D scores, and QLQ-BR23 scores for participants in terms such as frequency, mean, and standard deviation (SD). Inferential statistics applied Pearson’s correlation and linear regression analyzes.

Pearson’s correlation was used to determine the correlation between the scores of the HADS-A, HADS-D, and each dimension of QLQ-BR23, and linear regression analyses were performed to determine relationships between sociodemographic factors, support factors, treatments, and outcome variables.

In multiple linear regression analyses, outcome variables were the scores of psychological distress, and QLQ-BR23, all of which were continuous variables. Sociodemographic and support factors were considered independent variables. Age, duration of diagnosed breast cancer, and each support was a continuous variable. Dummy variables (categorization to zero and one) were marital status (married/lived together = 0, the others = 1), education (high school or above = 0, secondary school/others = 1), belonging to a cultural/ethnic minority (no = 0, yes = 1), having an underlying disease (no = 0, yes = 1), and methods of treatments (no = 0, yes = 1). Religion was excluded because of a low number in its subgroup. Assumptions were satisfied before the analyses (i.e., autocorrelation, multicollinearity, homoscedasticity, linearity, and multivariate normality). First, we inserted each independent variable into a simple linear regression analysis for each outcome variable. Significant independent variables from the simple analyses remained in multiple linear regression analyses using the stepwise selection method (alpha-to-enter of 0.05, alpha-to-remove of 0.10). We provided adjusted *R*^*2*^ and a standardized partial regression coefficient (*β*) and 95% confidence interval (CI) to demonstrate the fitness and strength of association of each outcome variable. The level of statistical significance for all analyses was set at *p* < 0.05.

## Results

The mean age (and SD) of participants was 62.7 (12.35), while the average number of year (and SD) from diagnosis was 2.9 (3.97). Approximately 60% of the participants had no underlying diseases, and 20% of them underwent breast reconstruction. See Table 1.

**Table 1:** Number and percentage of sociodemographic and treatment characteristics among participants (*n* = 481)

Characteristics	*n* (%)	
Age (years)^a^		
Mean = 62.7, SD = 12.35, Min = 31, Max = 93		
Civil status^a^		
Married/ Live together		321 (67.1)
Single/alone		157 (32.9)
Education level^a^		
Secondary school/unidentified	226 (47.4)	
High school or university	251 (52.6)	
Religion^a^		
Christian	422 (90.4)	
Non-Christian	45 (9.6)	
Cultural/Ethnic minority^a^		
No	455 (96.8)	
Yes	15 (3.2)	
Having an underlying disease^a^		
No		277 (59.3)
Yes		190 (40.7)
Duration from diagnosis (year)^a^		
Mean = 2.92, SD = 3.97, Min = 1, Max = 44		
Chemotherapy treatment^a^		
Yes		234 (49.6)
No		238 (50.4)
Radiation therapy treatment^a^		
Yes		227 (52.6)
No		252 (47.4)
Hormone therapy treatment^a^		
Yes		293 (62.7)
No		174 (37.3)
Herceptin treatment^a^		
Yes		82 (18.7)
No		357 (81.3)
Breast reconstruction^a^		
Yes		93 (19.7)
No		380 (80.3)
Total score of support from physicians^a^		
Mean = 6.36, SD = 2.78, Min = 0, Max = 9		
Total score of support from nurses^a^		
Mean = 4.60, SD = 3.22, Min = 0, Max = 9		
Total score of support from internet^a^		
Mean = 0.75, SD = 1.71, Min = 0, Max = 9		
Total score of support from husband/partner^a^		
Mean = 0.79, SD = 1.65, Min = 0, Max = 9		
Total score of support from family and friends^a^		
Mean = 0.77, SD = 1.56, Min = 0, Max = 9		
Total score of support from patient institution^a^		
Mean = 0.79, SD = 1.65, Min = 0, Max = 9		
HADS-A^a^		
Mean = 7.07, SD = 3.38, Min = 2, Max = 19		
HADS-D^a^		
Mean = 14.10, SD = 2.14, Min = 7, Max = 19		

^a^Obtained number < 481; Hospital Anxiety and Depression Scale – Anxiety sub-scale (HADS-A); Hospital Anxiety and Depression Scale – Depression sub-scale (HADS-D)

The correlation analyses demonstrated that the scores of HADS-A correlated significantly with all dimensions of the HRQoL except BRSEF and BRSEE, while HADS-D correlated significantly with all dimensions of the HRQoL. HADS-A had its strongest correlation 0.619 with BRFU, while HADS-D had its strongest correlation 0.325 with BRBI. See Table 2.

**Table 2:** Correlation between the scores of the Hospital Anxiety and Depression Scale – Anxiety sub-scale (HADS-A) and Depression sub-scale (HADS-D) and all dimensions of the Breast Cancer-Specific Quality of Life Questionnaire

Variables	BRBI	BRSEF	BRSEE	BRFU	BRST	BRBS	BRAS	BRHL
HADS-A	0.490*	0.067	0.130	0.619*	0.428*	0.330*	0.276*	0.171*
HADS-D	0.325*	0.159*	0.280*	0.316*	0.269*	0.256*	0.177*	0.104*

^*^Correlations were significant at 0.05 level

Body image (BRBI); sexual functioning (BRSEF); sexual enjoyment (BRSEE); future perspective (BRFU); systemic therapy side effects (BRST); breast symptoms (BRBS); arm symptoms (BRAS); and upset by hair loss (BRHL)

According to Table 3, breast cancer patients who were younger (*β* =  − 0.230, 95% CI − 0.180, − 0.279, *p* < 0.001) had an underlying disease (*β* = 0.219, 95% CI 0.118, 0.319, *p* < 0.001) and had received less support from physicians (*β* =  − 0.142, 95% CI − 0.212, − 0.071, *p* = 0.003) were likely to get increased symptoms of anxiety. Those who had an underlying disease (*β* = 0.116, 95% CI 0.072, 0.163, p = 0.015) and had undergone breast reconstruction (*β* = 0.116, 95% CI 0.061, 0.182, p = 0.013) were likely to get increased symptoms of depression.

**Table 3:** Multivariate linear regression analysis results of the scores of the HADS-A, HADS-D, and HRQoL

Variables	Unstandardized Coefficients			Standardized Coefficients		
	B	Standard error	95% CI	Beta (Descending)	t	p
HADS-A^a^						
Constant	9.447	1.008	7.466, 11.428		9.374	< .001*
Age	− 0.065	.014	− .092, − .038	− 0.230	− 4.692	< .001*
Having an underlying disease	1.526	.332	.874, 2.178	0.219	4.599	< .001*
Support from physicians	− 0.175	.059	− .291, − .059	− 0.142	− 2.965	.003*
HADS-D						
Constant	14.443	0.143	14.162, 14.723		101.289	< .001*
Breast reconstruction	0.626	0.252	.130, 1.121	0.116	2.482	.013*
Having an underlying disease	0.501	0.115	.099, .903	0.115	2.451	.015*
BRBI^a^						
Constant	59.467	6.742	46.211, 72.722		8.820	< .001*
Chemotherapy	8.027	2.301	3.503, 12.552	0.180	3.488	.001*
Having an underlying disease	7.761	2.278	3.283, 12.239	0.169	3.407	.001*
Age	− 0.303	.099	− .498, − .108	− 0.166	− 3.059	.002*
Breast reconstruction	5.589	2.758	.166, 11.011	0.102	2.026	.043*
BRSEF^a^						
Constant	57.389	4.227	49.079, 65.699		5.987	< .001*
Age	0.403	.067	.271, .536	0.281	5.987	< .001*
Having an underlying disease	5.530	1.643	2.299, 8.760	0.156	3.365	.001*
Support from husband/partner	− 1.532	.490	− 2.494, − .569	− 0.143	− 3.129	.002*
Belonging to a culture/ethnic minority	13.531	4.694	4.303, 22.759	0.131	2.882	.004*
BRSEE^a^						
Constant/	25.446	8.902	7.885, 43.006		2.859	.005*
Age	.535	0.161	.217, .853	0.247	3.316	.001*
Education	9.005	3.521	2.059, 15.951	0.191	2.558	.011*
Support from husband/partner	− 1.703	.789	− 3.259, − .147	− 0.145	− 2.159	.032*
BRFU^a^						
Constant	77.786	7.304	63.428, 92.144		10.649	< .001*
Age	− 0.548	0.097	− 0.739, − 0.357	− 0.272	− 5.639	< .001*
Having an underlying disease	11.497	2.251	7.072, 15.922	0.230	5.107	< .001*
Support from physicians	− 1.503	.402	− 2.293, − .713	− 0.171	− 3.741	< .001*
Chemotherapy	6.229	2.291	1.726, 10.732	0.127	2.719	.007*
BRST^a^						
Constant	28.431	1.578	25.328, 31.533		18.012	< .001*
Having an underlying disease	8.391	1.180	6.071, 10.711	0.316	7.110	< .001*
Chemotherapy	5.118	1.174	2.811, 7.424	0.197	4.361	< .001*
Support from family and friends	− 1.111	.366	− 1.830, − .393	− 0.135	− 3.040	.003*
Support from physicians	− .631	.212	− 1.048, − .215	− 0.135	− 2.979	.003*
BRBS^a^						
Constant	43.823	3.785	36.383, 51.262		11.579	< .001*
Radiotherapy	7.040	1.255	4.572, 9.507	0.252	5.607	< .001*
Belonging to a culture/ethnic minority	16.237	3.718	8.928, 23.545	0.195	4.367	< .001*
Age	− 0.226	0.054	− 0.332, − 0.119	− 0.196	− 4.159	< .001*
Having an underlying disease	4.680	1.304	2.118, 7.242	0.164	3.590	.018*
Support from nurses	− .477	.200	− .870, − .084	− 0.110	− 2.384	.018*
BRAS^a^						
Constant	28.234	1.265	25.746, 30.721		22.311	< .001*
Radiotherapy	8.455	1.661	5.190, 11.721	0.265	5.090	< .001*
Having an underlying disease	8.462	1.457	5.597, 11.326	0.260	5.808	< .001*
Chemotherapy	3.991	1.655	0.738, 7.244	0.125	2.411	.016*
BRHL						
Constant	32.794	1.879	29,100, 36.487		17.449	< .001*
Support from physicians	− .706	.271	− 1.238, − .174	− 0.123	− 2.608	.009*
Belonging to a culture/ethnic minority	10.450	4.430	1.744, 19.157	0.111	2.359	.019*

^*^A level of significance of 0.05

^a^Chemotherapy and support from nurses were significant only in univariate analyses for HADS-A; radiotherapy, Herceptin, support from physicians, and from nurses were significant only in univariate analyses for quality of life – body image. Breast reconstruction, civil status, education, and support from physicians were significant only in univariate analyses for BRSEF. Chemotherapy and support from internet were significant only in univariate analyses for BRSEE. Civil status and support from nurses were significant only in univariate analyses for BRFU. Age was significant only in univariate analysis for BRST. Chemotherapy, civil status, and support from physicians were significant only in univariate analyses for BRBS. Age and support from physicians were significant only in univariate analyses for BRAS

HADS-A, F = 17.551, *p* < .001, Adjusted *R*^*2*^ = 0.107

HADS-D, F = 5.585, *p* = .004, Adjusted *R*^*2*^ = 0.020

BRBI, F = 11.067, *p* < .001, Adjusted *R*^*2*^ = 0.093

BRSEF, F = 21.160, *p* < .001, Adjusted *R*^*2*^ = 0.164

BRSEE, F = 11.775, *p* < .001, Adjusted *R*^*2*^ = 0.146

BRFU, F = 24.916, *p* < .001, Adjusted *R*^*2*^ = 0.184

BRST, F = 23.228, *p* < .001, Adjusted *R*^*2*^ = 0.173

BRBS, F = 19.798, *p* < .001, Adjusted *R*^*2*^ = 0.185

BRAS, F = 27.954, *p* < .001, Adjusted *R*^*2*^ = 0.160

BRHL, *F* = 6.377, *p* = .002, Adjusted *R*^*2*^ = 0.024

Hospital, Anxiety, and Depression Scale – Anxiety subscale (HADS-A)**;** Hospital, Anxiety, and Depression Scale – Depression subscale (HADS-D)**;** Health-Related Quality of Life (HRQoL); Quality of life – body image (BRBI); Quality of life – sexual functioning (BRSEF); Quality of life – sexual enjoyment (BRSEE); Quality of life – future perspective (BRFU); Quality of life – systemic therapy side effects (BRST); Quality of life – breast symptoms (BRBS); Quality of life – arm symptoms (BRAS); and Quality of life – upset by hair loss (BRHL)

Patients with breast cancer who had been treated with chemotherapy (*β* = 0.180, 95% CI 0.145, 0.215, *p* = 0.001) and had an underlying disease (*β* = 0.169, 95% CI 0.136, 0.202, *p* = 0.001) were younger (*β* =  − 0.166, 95% CI − 0.265, − 0.067, *p* = 0.002) were associated with decreased HRQoL in BRBI. Patients who were older (*β* = 0.403, 95% CI 0.336, 0.470, *p* < 0.001) and had an underlying disease (*β* = 0.156, 95% CI 0.068, 0.244, *p* = 0.001) were associated with decreased HRQoL in BRSEF. Patients who were older (*β* = 0.247, 95% CI 0.162, 0.332, *p* = 0.001) and had low education (*β* = 0.191, 95% CI 0.090, 0.284, *p* = 0.011) were associated with decreased HRQoL in BRSEE. Patients who were younger (*β* =  − 0.272, 95% CI − 0.369, − 0.175, *p* < 0.001) and had an underlying disease (*β* = 0.230, 95% CI 0.196, 0.262, *p* < 0.001) were associated with decreased HRQoL in BRFU. Having an underlying disease (*β* = 0.316, 95% CI 0.282, 0.402, *p* < 0.001) and having undergone treatment with chemotherapy (*β* = 0.197, 95% CI 0.169, 0.232, *p* < 0.001) decreased HRQoL in BRST. Having received radiotherapy (*β* = 0.252, 95% CI 0.167, 0.336, *p* < 0.001) and belonging to an ethnic minority (*β* = 0.195, 95% CI 0.095, 0.289, *p* < 0.001) decreased HRQoL in BRBS. Having received radiotherapy (*β* = 0.265, 95% CI 0.180, 0.348, *p* < 0.001) and having an underlying disease (*β* = 0.260, 95% CI 0.174, 0.342, *p* < 0.001) were factors associated with decreased HRQoL in BRAS. Patients who had received increased support from physicians (*β* = 0.123, 95% CI 0.108, 0.138, *p* = 0.009) and belonged to an ethnic minority (*β* = 0.111, 95% CI 0.096, 0.127, *p* = 0.019) were associated with decreased HRQoL in BRHL.

## Discussion

In this study, both anxiety and depressive symptoms demonstrated the highest correlation with HRQoL in the dimensions of future perspective and body image. Psychological distress is commonly diagnosed among patients with breast cancer [7, 30]. People living with psychological distress may experience an imbalance between their realities and their ideal wishes, resulting in a breakdown in their self-esteem and low well-being [9]. Moreover, psychological distress is recognized as associated with decreased HRQoL among patients [31]. Greater depressive symptoms are associated with more emotional suppression [12], and suicidal thoughts, and attempted suicide may occur among women with breast cancer suffering from depressive symptoms [11]. It might be useful to examine psychological distress and HRQoL along with treatment of psychologically vulnerable women, like women with breast cancer.

Our study revealed that participants who had an underlying disease were more likely to have psychological distress. Moreover, those having breast reconstruction might have more symptoms of psychological distress. People with psychological distress seem to have reduced capacity and lack of control of their everyday lives [9]. Although having an underlying disease is common, comorbidity can make life difficult for patients with breast cancer [32]. Also, after life change events like surgery, patients with breast cancer may experience psychological distress [12]. Therefore, a preventive intervention related to emotional awareness for such patients with breast cancer should be implemented. For instance, the mindfulness-based stress reduction program has potential to improve the mental health among women with breast cancer [2].

External sources, like support from healthcare professionals (HCPs), could reduce psychological distress [33], while poor support contributes to psychological distress [34]. The development of treatment plans by physicians and patients is essential [35]. Patients need to be involved in a person-centered dialogue with physicians to strengthen their own capacities for daily lives [9]. Therefore, our study suggests that HCPs should provide sufficient information to patients with breast cancer and include the patients in their planning. This may contribute to decreased psychological distress.

Several sources of support (e.g., physicians, nurses, and husband/partner) have been found to be related to decreased HRQoL for many dimensions. The participants needed support from their husbands/partners about their sexual functioning and enjoyment. After treatments of breast cancer, sexual dysfunction becomes a challenge for patients [35]. They need more support and tenderness from their partners [32] to maintain their HRQoL [35]. Patients with breast cancer in our study also thought about side effects of their therapy, e.g., breast symptoms and hair loss. Moreover, those treated with radiotherapy and chemotherapy needed information from HCPs about their current and future lives to increase their well-being. Cognitive behavioral therapy and supportive-expressive group therapy give positive effects on patients with breast cancer [36]. Thus, after treatments of patients with breast cancer, individual or group therapy may assist the improvement of the HRQoL among the patients.

## Strengths and limitations

We constructed the DAGs from reviewed literature, which assisted data collection and analyses. Some confounders, such as age, had been adjusted by multiple linear regression analyses. The use of real scores from the questionnaire rather than categorized scores enhanced estimates [37]. In addition, all instruments used in this study had been tested before data collection with acceptable values of validity and reliability.

The cross-sectional character of the study limited cause-effect relationships. Therefore, subsequent longitudinal studies may more clearly explain factors associated with psychological distress and health-related quality of life. Some information bias could be seen because the participants self-reported. Moreover, some information that might be related to psychological distress and HRQoL (e.g., pathological stage and metastasis) had not been collected. In addition, the rate of participation was only 60% which may also have affected our findings.

## Conclusions

Psychological distress was correlated with most dimensions of HRQoL. The strongest correlation was found for anxiety symptoms and future perspective and depressive symptoms and body image. Women with breast cancer who were younger were likely to get increased symptoms of anxiety, while those who had undergone breast reconstruction were likely to get increased symptoms of depression. Low support from HCPs decreased the HRQoL in terms of future perspective, systemic therapy side effects, breast symptoms, and indignation about hair loss. Support from husband/partner increased the HRQoL in terms of sexual functioning and enjoyment. Treatment with chemotherapy decreased the HRQoL in terms of body image, systemic therapy side effects, and arm symptoms. Women with breast cancer need support from many sources, in particular HCPs and their husbands/partners.

## Data Availability

The datasets used in the current study are available from the corresponding author on reasonable request.
